# Prediction and Classification of COVID-19 Admissions to Intensive Care Units (ICU) Using Weighted Radial Kernel SVM Coupled with Recursive Feature Elimination (RFE)

**DOI:** 10.3390/life12071100

**Published:** 2022-07-21

**Authors:** Huda M. Alshanbari, Tahir Mehmood, Waqas Sami, Wael Alturaiki, Mauawia A. Hamza, Bandar Alosaimi

**Affiliations:** 1 Department of Mathematical Sciences, College of Science, Princess Nourah bint Abdulrahman University, P.O. Box 84428, Riyadh 11671, Saudi Arabia; hmalshanbari@pnu.edu.sa; 2 School of Natural Sciences (SNS), National University of Sciences and Technology (NUST), Islamabad 44000, Pakistan; tahime@gmail.com; 3 Department of Community Medicine and Public Health, College of Medicine, Majmaah University, Majmaah 11952, Saudi Arabia; w.mahmood@mu.edu.sa; 4 Azra Naheed Medical College, Superior University, Lahore 54000, Pakistan; 5 Department of Medical Laboratory Sciences, College of Applied Medical Sciences, Majmaah University, Majmaah 11952, Saudi Arabia; w.alturaiki@mu.edu.sa; 6 Faculty of Medicine, King Fahad Medical City, Riyadh 11525, Saudi Arabia; mahahmed@kfmc.med.sa; 7 Research Center, King Fahad Medical City, Riyadh 11525, Saudi Arabia

**Keywords:** healthcare systems, machine learning, support vector machine, COVID-19 burden, classification, prediction, ICU, public health measures

## Abstract

Healthcare systems have been under immense pressure since the beginning of the COVID-19 pandemic; hence, studies on using machine learning (ML) methods for classifying ICU admissions and resource allocation are urgently needed. We investigated whether ML can propose a useful classification model for predicting the ICU admissions of COVID-19 patients. In this retrospective study, the clinical characteristics and laboratory findings of 100 patients with laboratory-confirmed COVID-19 tests were retrieved between May 2020 and January 2021. Based on patients’ demographic and clinical data, we analyzed the capability of the proposed weighted radial kernel support vector machine (SVM), coupled with **(RFE)**. The proposed method is compared with other reference methods such as linear discriminant analysis (LDA) and kernel-based SVM variants including the linear, polynomial, and radial kernels coupled with REF for predicting ICU admissions of COVID-19 patients. An initial performance assessment indicated that the SVM with weighted radial kernels coupled with REF outperformed the other classification methods in discriminating between ICU and non-ICU admissions in COVID-19 patients. Furthermore, applying the Recursive Feature Elimination (RFE) with weighted radial kernel SVM identified a significant set of variables that can predict and statistically distinguish ICU from non-ICU COVID-19 patients. The patients’ weight, PCR Ct Value, CCL19, INF-β, BLC, INR, PT, PTT, CKMB, HB, platelets, RBC, urea, creatinine and albumin results were found to be the significant predicting features. We believe that weighted radial kernel SVM can be used as an assisting ML approach to guide hospital decision makers in resource allocation and mobilization between intensive care and isolation units. We model the data retrospectively on a selected subset of patient-derived variables based on previous knowledge of ICU admission and this needs to be trained in order to forecast prospectively.

## 1. Introduction

The severe acute respiratory syndrome coronavirus 2 (SARS-CoV-2) that causes coronavirus disease (COVID-19) was first detected in Wuhan, China, in December 2019 [1]. The virus was isolated from throat swab samples from patients with clinical signs of dry cough, dyspnea, fever, and bilateral lung infiltrates [2]. The majority of COVID-19-affected patients experienced only minor symptoms such as a dry cough, sore throat, and fever. However, some also died due to fatal complications such as heart failure, septic shock, pulmonary edema, pneumonia, and acute respiratory distress [3].

According to the emergency committee, the spread of COVID-19 could be slowed by early diagnosis, isolation, timely care, and the introduction of a comprehensive mechanism for tracing contacts [3]. To mitigate the surge of the pandemic, most countries focused on isolation, quarantine, social distancing, and infection containment. By mid-January 2022, over 327 million confirmed COVID-19 cases and more than 5.5 million deaths were recorded across the globe. Saudi Arabia reported more than 600 thousand confirmed cases and nearly 9000 deaths during the same period [4].

Healthcare systems have been under immense pressure since the beginning of the COVID-19 pandemic; hence, studies on using machine learning (ML) methods for classifying ICU admissions and resource allocation are urgently needed. Numerous studies show that demographic data, laboratory parameters, and clinical characteristics may aid in classifying COVID-19 severity levels. For example, differences in cross-country fatalities related to COVID-19 were recognized by the importance of the population age structure and intergenerational connections [5]. In a longitudinal descriptive cohort study, the analyses of complete blood count parameters, patient age, gender, and co-morbidities suggested the use of patient demographics and management outcomes as predictive clinical biomarkers [6]. Chest CT imaging has been used as a means of determining COVID-19 severity among symptomatic high-risk individuals [7]. Immune biomarkers’ regulations in COVID-19 patients have been associated with disease severity, complications, and mortality rate [8]. COVID-19-positive ICU patients had a significant increase in blood coagulation abnormality, which was associated with many inflammatory up/down regulations [9] and cardiac complications such as acute myocardial infarction [10]. High levels of liver enzymes such as alanine transaminase (ALT) and aspartate aminotransferase (AST) have been associated with lengthy periods of hospitalization in COVID-19 patients [11]. Kidney diseases have also been linked to COVID-19 severity [12].

These reviewed studies used univariate or bi-variate analysis to investigate the impact of one or more factors on COVID-19 severity. Several multivariate machine learning techniques [13,14,15,16] exist; among these are Linear discriminant analysis (LDA) and support vector machine (SVM), which are considered to have a potential predicting power [17,18]. Therefore, the main aim of this article is to predict the admission of COVID 19-patients in ICU based on related factors through weighted radial kernel-based SVM coupled with Recursive Feature Elimination (RFE) methods. The standard SVM method uses a linear kernel approach, whereas the latest variants comprise polynomial, radial, weighted, and Gaussian kernels within SVM. The implementation of Recursive Feature Elimination (RFE) over these kernels (polynomial, radial, and Gaussian) in SVM has been proposed as a classification tool for dermoscopic images of skin cancer patients [19]. We have implemented REF with weighted radial kernel SVM and compared it with other SVM variants and standard ML methods, such as LDA, for predicting ICU admission among COVID-19 patients. The application of SVM in medical science is novel, where ML methods can be used for classifying ICU admissions and resource allocation with less human involvement and with higher accuracy. This study proposed the development of an SVM model that can assess the clinical severity of COVID-19 and improve hospitalization preparedness by assorting and predicting the need for ICU admission in COVID-19 patients.

## 2. Materials and Methods

### 2.1. COVID-19 Data Set

The study data set of 100 patients was collected retrospectively from a population who were admitted to Prince Mohammed Bin Abdulaziz Hospital, Saudi Arabia, between May 2020 and January 2021. There were 50 critical cases that required ICU admission, and 50 mild cases (non-ICU). Ten individuals died (all of them were admitted to the ICU), while the others survived. Fifty-seven patients were Saudi citizens. Descriptions of relevant demographic and clinical characteristics are displayed in Table 1.

The data set comprised socio-demographic data such as gender, age, and nationality, and risk markers included features such as respiratory disease data, as well as chronic, circulatory, metabolic, and kidney diseases. We also retrieved the data, D-dimar, CK, CKMB, troponin, complete blood parameters (RBCs, WBCs, platelets, HB, neutrophils, monocytes, eosinophils, basophiles, and Lymphocytes), glucose, CRP, ESR, AST, ALT, albumin, urea, total protein, creatinine, INR, PT, PTT, and LDH. We also included immune markers in the analysis (BLYS, TNFSF13, BLC, C-C motif chemokine ligand 19, SDF-1, c-c motif chemokine ligand 21, and interferon IFN-β). Table 1 shows the distribution of demographic and clinical characteristics of the ICU and non-ICU patients. The protocol was approved by PNU Institutional Research Board (IRB); the approval number was 21-0309. Hence, written informed consent was waived and not required, since only unidentifiable data were extracted from the medical records.

### 2.2. Classification Methods

Linear discriminant analysis (LDA) and support vector machines (SVM) with linear, polynomial, radial, and weighted radial kernels were implemented to discriminate between ICU and non-ICU in COVID-19 patients based on several socio-demographic and risk factors. The consistency index was used with kernel support-vector machines (SVM) for the identification of associated markers.

### 2.3. Linear Discriminant Analysis (LDA)

LDA is a fundamental statistical discrimination method that assumes that the data matrix\bm{X} against ICU and non-ICU classes follows multivariate normal distribution and is assumed to be homogeneous for both ICU and non-ICU classes. In LDA, the decision boundary for classes is determined by the following:

$$\delta_{c\left(x\right)}=-\left(1/2\right)log\left|\sum \right|-1/2{\left(x-\mu_{c}\right)}^{\prime }\sum^{-1}\left(x-\mu_{c}\right)+log\pi_{c}$$

where ∑ is the variance-covariance matrix of X, $\mu_{c}$ is the mean vector of dX for class c, and $\pi_{c}$ includes the prior probabilities, which usually comprise the size proportion of the respective class c. In this study, $\pi_{c}$ is taken as 0.5.

### 2.4. Support Vector Machine (SVM)

For the classification of ICU admissions, SVM creates a hyperplane in high-dimensional space, which is a group of points in a data matrix X that meet the following criteria:

$$\vec{w}\cdot \vec{x}-b=0,$$

where $\vec{w}$ is the normal vector to the hyperplane. Parameter $\frac{b}{∥\vec{w}∥}$ calculates the usual vector offset of the hyperplane from the origin. The dot product between two feature vectors in a Hilbert space is computed by a kernel-based SVM, which opens up a new horizon for classification.

The linear kernel is used in [20], polynomial kernel in [21], radial, also called Gaussian, kernel in [22], and weighted radial kernel in [23].

The linear kernel is defined as follows.

$$k\left(x_{i},x_{j}\right)=x.x_{j}$$

The polynomial kernel is defined as follows:

$$k\left(x_{i},x_{j}\right)={\left(x_{i}.x_{j}+1\right)}^{d}$$

where d is the degree of polynomial.

The radial kernel, also called the Gaussian kernel, is defined as follows:

$$k\left(x_{i},x_{j}\right)=e^{-\frac{|x_{i},x_{j}{|}^{2}}{2\sigma^{2}}}$$

where x is risk factor or socio-demographic variable and σ is the standard deviation.

The weighted radial kernel is defined as follows:

$$k\left(x_{i},x_{j}\right)=e^{-w\frac{|x_{i},x_{j}{|}^{2}}{2\sigma^{2}}}$$

where *x* is the risk factor or socio-demographic variable, σ is the standard deviation, and w is the weight of the radial kernel.

### 2.5. Recursive Feature Elimination (RFE) in SVM

A feature selection, i.e., factor selection, procedure was adopted here to eliminate the irrelevant factors used in ICU and non-ICU discrimination. The recursive feature elimination (RFE) method [24] is used with SVM. The influential factors are recognized by a signal-to-noise ratio through orthogonal arrays. REF is convenient in hospital settings and considered as a variant of backward feature elimination. The implementation of RFE over these kernel (polynomial, radial, and Gaussian) SVM is the part of latest research. We have implemented REF to weighted radial kernel SVMs, which outperforms other SVM variants and standard methods for predicting ICU admissions among COVID-19 patients. Compared to traditional feature selection methods, REF not only improves the model accuracy on test data but also assists in understanding the ICU and non-ICU admission requirements.

### 2.6. Repeated Cross-Validated Accuracy

ICU admissions classifiers need to be validated, which constitutes how well the ICU classifiers can classify a new person into ICU or non-ICU admissions.

Accuracy is defined as follows.

$$\mathrm{Accuracy}=\frac{\mathrm{Number}\;\mathrm{of}\;\mathrm{correct}\;\mathrm{ICU}\;\mathrm{predictions}}{\mathrm{Total}\;\mathrm{number}\;\mathrm{of}\;\mathrm{predictions}}$$

It ranges from 0% to 100%, and a high percentage indicates that the respective ICU admission classifier is preferable. For validation, several schemes exist. We have used 20-fold cross-validation repeated 10 times. The 20-fold cross-validation contains 20 iterations, and in each iteration, the ICU admissions response y and the data matrix X is divided into 20 folds. In the first iteration except the first fold, the remaining nine folds are used to train the ICU classifiers. The accuracy of the trained classifier is measured on test data. At second iteration, the validated accuracy of the classifier is measured from the second fold and so on. Lastly, the accuracy of the respective classifier from each iteration is averaged for that specific run.

## 3. Results

This section is divided by subheadings and presents a concise and precise description of the experimental results and their interpretation, and the experimental conclusions that have been drawn.

**Figure 1:**
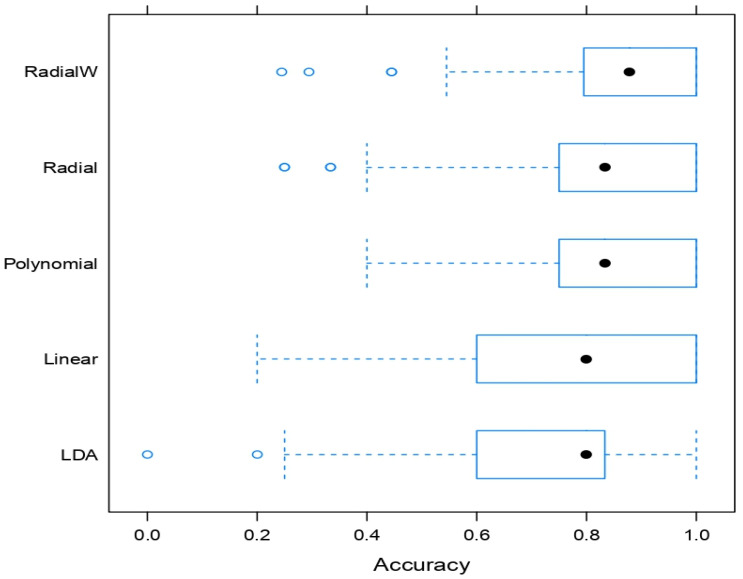
The comparison of validated accuracies of ICU and non-ICU discrimination including LDA, linear, polynomial, radial, and weighted radial SVM.

To model the COVID-19 severity, 44 predicting features were used based on the patient’s medical information, including respiratory, chronic, circulatory, metabolic, and kidney diseases, PCR Ct values, ICU admission requirements, clot formations, cardiac enzymes, troponin tests, hematology profiles, sugar levels, and liver and kidney tests. By applying repeated 20-fold cross-validation, the ICU discrimination classifiers, including LDA, linear, polynomial, radial, and weighted radial SVM, were trained. The comparison of the validated accuracy of these classifiers is presented in Figure 1. It was found that LDA method classified 80% of patients as requiring admission to ICU. The patients’ weight, PCR Ct value, CCL19, INF-β, BLC, INR, PT, PTT, CKMB, HB, platelets, RBC, urea, creatinine, and albumin results were found to be the predicting features. The average validated accuracies of LDA and linear SVM were similar at around 80%. LDA showed less variation in the validated accuracy as compared to linear SVM. SVM with polynomial and radial kernel functions in its standard form classified 83% patients as requiring admission in ICU or not, while its advanced variant, SVM weighted radial kernel function, classified 88% of the patients as ICU- or non-ICU-admitted patients. This indicated that the weighted radial kernel SVM best discriminated among the ICU and non-ICU admission of patients.

**Figure 2:**
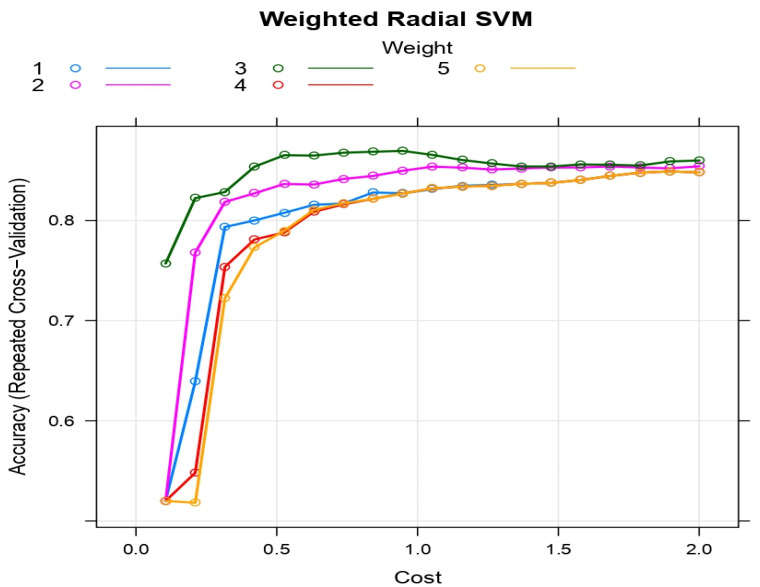
The repeated 20-fold cross-validation accuracies are presented at different values in terms of cost and weight.

The weighted radial kernel SVM is characterized by two tuning parameters: the regularization parameter (cost) and the weights that can be optimized by grid searching. Let cost = (0, 0.2, 0.4, …, 2) and weight = (1, 2, 3, 4, 5); then, the repeated 20-fold cross-validation was used at each combination of cost and weight. The average validated accuracy increases with the increase in cost levels. The average validated accuracy is optimized with weights = 3. As a result, the weighted radial SVM is optimized with weight = 3 and cost = 0.6. The repeated 20-fold cross-validation accuracy is presented in Figure 2 for different values of cost and weight of weighted radial SVM.

**Figure 3:**
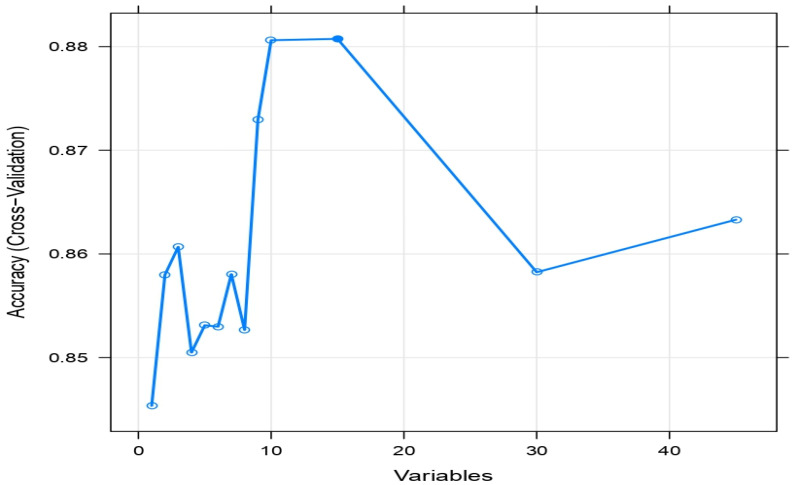
The cross-validated accuracy of ICU patients extracted by RFE weighted radial SVM models.

Recursive feature elimination (RFE) is employed over the weighted kernel SVM for the identification of influential factors that discriminate between ICU and non-ICU patients. To acquire insight into how RFE optimizes the weighted radial SVM models, the cross-validated accuracy of ICU patients at each backward step of RFE is presented in Figure 3. This indicates that the optimal weighted radial SVM model selects 15 and 10 risk factors with an optimal accuracy of 88.1%. Since our interest is to have a better understanding of the fitted model where more factors can be included in the model, we have considered the optimal model with 15 risk factors. The influential factors that can discriminate between ICU and non-ICU patients with COVID-19 are weight, CT, CCL19, INF-β, BLC, INR, PT, PTT, CKMB, HB, platelet, RBC, urea, creatinine, and albumin, which are presented in Table 2.

## 4. Discussion

In this study, we propose the capability of several ML methods to identify the predicting features that can be extrapolated by modelling the incremental patient data, thus providing optimal estimators for forecasting the need of ICU admissions. Based on the analysis in a cohort with 100 SARS-CoV-2-positive patients in admissions to ICU and non-ICU, we found that, out of five ML methods in stratifying and predicting the need of different levels of hospital care, the SVM with weighted radial kernel outperformed other classification methods using the demographic and clinical parameters.

We applied LDA, linear SVM, polynomial SVM, radial SVM, and weighted radial SVM with 20-fold-cross-validation. Weighted radial SVM outperformed the other methods by achieving an accuracy of 88.1%. Pourhomayoun and Shakibi [25] used laboratory data to identify patients who require special care and to forecast durations of stay in specialized care units. Similar to our approach, the authors trained numerous machine learning algorithms to estimate the mortality rate of COVID-19 patients, including support vector machines, neural networks, random forest, decision tree, logistic regression, and k-nearest neighbors. Their results indicated that the neural network method performs best, with an accuracy of 93.75%.

Our study indicates that the weight of the patient is a significant factor that can discriminate between a patient’s admission into ICU or not among COVID-19 patients. With increase in weight, a COVID-19 patient is more likely to be admitted to the ICU. This study considers the PCR Ct value as a predicting feature. With the decrease in Ct value, the chances of ICU admission increase. This was also observed in [7], where the Ct-values of real-time RT-PCR assay decrease as the patients recover. The weight of COVID-19 patients is an influential factor that can discriminate between ICU and non-ICU patients with COVID-19. The patients admitted in ICU had an average weight of 95.4 kg, while non-ICU patients had an average weight of 81.8 kg.

The optical densities of BLYS, TNFSF13, BLC, SDF-1, C-C MOTIF CHEMOKINE LIGAND 21, MIP-3-BETA, and IFN-β are prognostic biomarkers for the COVID-19 immune response. Among these biomarkers, MIP-3-BETA, IFN-β, and BLC were found to be predicting factors, where the upregulation of these biomarkers will increase the patients’ chances of requiring ICU admission. Similar findings were observed in [8], where patients with COVID-19 experienced the activation of the complement system, which was attributed to disease severity.

We have shown that SVM with weighted radial kernel was capable of classifying patients, with high accuracy, into two categories: ICU and non-ICU COVID-19 patients. Blood coagulation factors (INR, PT, and PTT) were significant predictors in our study, as well as in previous studies [9] where blood coagulation disorder was prominent in ICU patients with COVID- 19 and was correlated with multi-inflammation factors. Similarly, the cardiac enzymes (CK and CK-MB) were CK-MB-significant indicators for ICU admission and COVID-19-associated acute myocardial infarction [10]. To identify COVID-19 patients at risk of deterioration during hospitalization using complete blood count, we observed that the decrease in HB, platelets, and RBC and the increase in WBC were classifiers to predict patients at the highest risk, which was also observed in [6].

Gu and colleagues [11] used elevated serum AST levels to identify patients requiring special care and to predict the lengths of stay at ICU units. The authors tested several ML algorithms to select the best performance. Similar to our study, the authors used laboratory data and found an association between length of hospital stay in COVID-19 patients and elevated levels of serum AST. Creatinine test was also used in previous studies for predicting ICU admissions of COVID-19 patients [12], and in our study, we also found it to be an influential indicator for ICU admission.

The current study proposes a clinically interpretable model for classifying the ICU and non-ICU of COVID-19 patients. However, the reported results are limited to the representative size of the sampled population. Findings may vary according to various factors such as location and culture. Furthermore, we model data retrospectively based on a selected subset of patient-derived variables with previous knowledge of ICU admission and the need to be trained to forecast prospectively.

## 5. Conclusions

We applied kernel-based SVM algorithms to predict ICU admission of COVID- 19 patients. The weighted kernel SVM outperformed other classifiers by achieving an accuracy of 88.1%. The algorithm also identified 15 influential factors: The patients’ weight, PCR Ct value, CCL19, INF-β, BLC, INR, PT, PTT, CKMB, HB, platelets, RBC, urea, creatinine, and albumin results were found to be the predicting features. We believe that our findings constitute the usability of machine learning to provide optimal estimators for predicting outcomes in the current pandemic, to enable new perspectives in decision making, and pave the way to determining healthcare priorities with real-time assessments.

## Figures and Tables

**Table 1:** The distribution of demographic and clinical characteristics of the ICU and non-ICU patients.

Variable	All Patients (*n* = 100)	Non-ICU (*n* = 50)	ICU (*n* = 50)
Demographic			
Age			
range	24–84	24–84	25–79
Gender			
Male	56 (56%)	25 (50%)	31 (62%)
Female	44 (44%)	25 (50%)	19 (38%)
Nationality			
Saudi	57 (57%)	28 (56%)	29 (58%)
Non- Saudi	43 (43%)	22 (44%)	21 (42%)
Fatality			
Died	10 (10%)	0 (0%)	10 (20%)
Survived	90 (90%)	50 (100%)	40 (80%)
Respiratory Disease			
Yes	12 (12%)	3 (6%)	9 (18%)
No	88 (88%)	47 (94%)	41 (82%)
Chronic Disease			
Yes	62 (62%)	30 (60%)	32 (64%)
No	38 (38%)	20 (40%)	18 (36%)
Circulatory Disease			
Yes	47 (47%)	24 (48%)	23 (46%)
No	53 (53%)	26 (52%)	27 (54%)
Metabolic Disease			
Yes	62 (62%)	20 (40%)	18 (36%)
No	38 (38%)	30 (60%)	32 (64%)
Kidney Disease			
Yes	8 (8%)	1 (2%)	7 (14%)
No	92 (92%)	49 (98%)	43 (86%)

**Table 2:** The optimal weighted radial SVM model. The SVM model predicted 15 risk factors with significant *p*-values discriminating ICU patients from non-ICU patients.

	Variable	Non-ICU Mean (SD)	ICU Mean (SD)	*p*-Value *
1	Weight	81.8 (18.6)	95.4 (30.9)	0.009
2	PCR Ct Value	27.0 (4.1)	25.4 (5.3)	0.091
3	CCL19	0.1 (0.2)	0.2 (0.2)	0.307
4	INF-β	12.8 (21.7)	53.4 (55.1)	<0.001
5	BLC	0.2 (0.2)	0.5 (0.6)	0.001
6	INR	1.0 (0.2)	1.3 (0.8)	0.009
7	PT	13.6 (2.3)	17.6 (10.4)	0.009
8	PTT	38.0 (10.7)	45.3 (13.4)	0.003
9	CK.MB	26.1 (8.4)	43.8 (58.5)	0.036
10	HB	12.6 (2.0)	9.5 (2.1)	<0.001
11	Platelets	346.3 (118.8)	239.5 (132.3)	<0.001
12	RBC	4.5 (0.6)	3.3 (0.8)	<0.001
13	Urea	6.3 (3.4)	13.5 (11.1)	<0.001
14	Creatinine	78.5 (28.9)	116.0 (85.1)	0.004
15	Albumin	33.4 (3.8)	27.1 (6.0)	<0.001

* Significant at *p*-value > 0.05.

## Data Availability

The data sets generated during and/or analyzed during the current study are available from the corresponding author upon reasonable request.
